# Thyroid dysfunction in non-alcoholic fatty liver disease: A case-control study

**DOI:** 10.6026/973206300212453

**Published:** 2025-08-31

**Authors:** Harshit Malkan, Bhavita Patel, Mahesh G. Solu, Tejas D. Patel, Mahammed Kaif Mubrakhusen Bhaniya

**Affiliations:** 1 Department of General Medicine, Dr. Kiran C Patel Medical College and Research Institute, Bharuch, Gujarat, India; 2 Department of Biochemistry, Dr. Kiran C Patel Medical College and Research institute, Bharuch, Gujarat, India; 3 Department of Orthopaedic Surgery, Dr. Kiran C Patel Medical College & Research Institute, Bharuch, Gujarat, India; 4 Trauma Centre, Dr Kiran C Patel Medical College and Research institute, Bharuch, Gujarat, India

**Keywords:** Metabolic dysfunction, non-alcoholic fatty liver disease (NAFLD), thyroid dysfunction

## Abstract

Non-alcoholic fatty liver disease (NAFLD) is increasingly recognized as a hepatic manifestation of metabolic dysfunction, with emerging evidence linking it to thyroid abnormalities. In this case-control study of 120 adults, thyroid dysfunction was more prevalent in NAFLD cases, with 20% having overt hypothyroidism and 5% subclinical hypothyroidism. Thyroid dysfunction increased with NAFLD severity, with overt hypothyroidism observed in 66.7% of Grade 3 cases. These findings highlight a strong association between worsening thyroid function and NAFLD severity, suggesting the need for routine thyroid screening in NAFLD management.

## Background:

Non-alcoholic fatty liver disease (NAFLD), affecting around 25% of the global population, is characterized by hepatic fat accumulation unrelated to significant alcohol intake and is closely linked to obesity and diabetes [1]. It ranges from simple steatosis to non-alcoholic steatohepatitis (NASH), with potential progression to cirrhosis and hepatocellular carcinoma [1]. Insulin resistance, oxidative stress, lipid metabolism abnormalities and genetic factors contribute to its pathogenesis [2]. Recent studies have highlighted a significant association between thyroid dysfunction and NAFLD [3]. Thyroid hormones, particularly triiodothyronine (T3) and thyroxine (T4), regulate hepatic lipid and carbohydrate metabolism through the thyroid hormone receptor beta (TRβ) [4]. Hypothyroidism contributes to metabolic disturbances such as insulin resistance and dyslipidemia, which may exacerbate NAFLD [3]. Low free T3 (FT3) levels have been inversely correlated with NAFLD severity, indicating thyroid dysfunction as a potential marker of disease progression [5]. Thyroid hormone receptor agonists like resmetirom are under investigation for therapeutic benefits in reducing hepatic inflammation and fibrosis in NAFLD [4]. Recent meta-analyses have strengthened the evidence linking thyroid dysfunction to NAFLD. Eshraghian *et al.* [6] reviewed 11 studies (n=12,924), highlighting a high prevalence of both overt and subclinical hypothyroidism in NAFLD/NASH, albeit with inter-study variability. Gariani *et al.* [7] confirmed a significant association between overt hypothyroidism and NAFLD risk, along with elevated TSH levels in NAFLD patients. Li *et al.* [8] further demonstrated a dose-dependent relationship, showing that higher free T3 increases NAFLD risk, whereas elevated free T4 offers a protective effect. Therefore, it is of interest to investigate the relationship between thyroid dysfunction and the severity of NAFLD, providing insights into the underlying mechanisms and potential clinical implications.

## Materials and Methods:

## Study design and participants:

This case-control study was conducted to evaluate the association between non-alcoholic liver disease (NAFLD) and thyroid dysfunction, metabolic parameters and liver function markers. A total of 120 participants in the age group of above 18 years were included, comprising 60 patients diagnosed with NAFLD (cases) and 60 healthy individuals (controls). The study was conducted at Outpatient departments (OPD) and intensive care units (ICUs) of Department of Medicine, Civil Hospital, Bharuch managed by Dr. Kiran C Patel Medical College and Research Institute for period of 6 months [August 2024 to February 2024]. Ethical approval was obtained from the institutional ethics committee IEC/2024/27, Dated 24th July 2024] and written informed consent was obtained from all participants.

## Inclusion criteria:

Adults aged ≥18 years, diagnosed with NAFLD were included in the case group. The presence of NAFLD defined as hepatic steatosis in the absence of excessive alcohol consumption or previous liver disease was based on the ultrasound evaluation, according to standardized criteria [9]. Healthy individuals without any chronic or acute illness were included as controls.

## Exclusion criteria:

Patients who is a known case of intrinsic thyroid disorders and other liver disorders, patients with hypothalamus and pituitary gland dysfunction and patients on drugs that affect thyroid hormone levels such as inorganic iodine, iodide, amiodarone, cyanates, lithium, radio contrast material containing iodine, tyrosine kinase inhibitors, interferon alpha, interleukin 2 *etc.*, were excluded from the study. Individuals suffering from any form of chronic or acute illness were excluded from the study.

## Data collection and clinical assessment:

Patient history, including demographics, past medical history, family medical history, medication history, comorbidities, substance abuse were compiled and documented using a standardized interview. Anthropometric measurements, including body mass index (BMI) and waist circumference, were obtained using standardized protocols. BMI was classified according to the World Health Organization (WHO) criteria, with obesity defined as BMI ≥ 25 kg/m^2^.

## Diagnosis and grading of NAFLD:

The presence and severity of NAFLD were assessed using ultrasonography. NAFLD was graded into three categories: Grade 1 (mild), Grade 2 (moderate) and Grade 3 (severe) based on liver echotexture, attenuation and visualization of hepatic vasculature.

## Biochemical and laboratory investigations:

Under aseptic conditions, venous blood samples of about 5 ml to be collected by venipuncture from Medial Cubital Vein was used for the assessment investigations at the time of admission. The following parameters were measured:

[1] Liver function tests: The Mindray automatic biochemistry analyser BS430 was used for serum glutamate pyruvate transaminase (SGPT), serum glutamate oxaloacetate transaminase (SGOT), total bilirubin, direct bilirubin and indirect bilirubin.

[2] Thyroid function tests: The Mindray automatic Electrochemiluminescence biochemistry analyser CL900i was used for Thyroid-stimulating hormone (TSH), free triiodothyronine (fT3) and free thyroxine (fT4). Thyroid dysfunction was classified as normal thyroid function, overt hypothyroidism and subclinical hypothyroidism.

[3] Metabolic parameters: Fasting blood glucose, glycated haemoglobin (HbA1c), lipid profile (total cholesterol, LDL-c, HDL-c and triglycerides).

[4] Haematological parameters: Haemoglobin (Hb), mean corpuscular volume (MCV) and mean corpuscular haemoglobin (MCH).

## Statistical analysis:

Data were analysed using SPSS version 20. Continuous variables were expressed as mean ± standard deviation (SD) and compared using an independent t-test or one-way ANOVA, as appropriate. Categorical variables were presented as frequencies and percentages and analysed using the chi-square test. A p-value < 0.05 was considered statistically significant.

## Results:

The study included 60 patients diagnosed with non-alcoholic liver disease and 60 healthy controls. The mean age was comparable between cases (40.7 ± 12.93 years) and controls (44 ± 16.04 years, p = 0.217). Males predominated in both groups (76.7% vs. 60%, p = 0.070). Cases had significantly higher BMI (27.8 ± 4.6 vs. 24.5 ± 3.8 kg/m^2^, p = 0.002) and waist circumference (96.8 ± 10.5 vs. 85.4 ± 9.2 cm, p < 0.001), with obesity (BMI ≥ 25 kg/^2^) more common in cases (50% vs. 33.3%). Diabetes (36.7% vs. 13.3%, p = 0.005) and hypertension (43.3% vs. 20%, p = 0.012) were also significantly higher among cases. Smoking was more frequent in cases (30% vs. 16.7%, p = 0.093) but not statistically significant. Among NAFLD patients, 45% had Grade 1, 35% had Grade 2 and 20% had Grade 3 fatty livers (Table 1 - see PDF). Thyroid dysfunction was significantly more prevalent in NAFLD patients than controls (p < 0.001). Among cases, 25% had thyroid dysfunction (20% overt and 5% subclinical hypothyroidism), while all controls had normal thyroid function. Mean TSH was higher in cases (5.38 ± 5.71 vs. 2.73 ± 1.47 µIU/mL, p < 0.001), with elevated TSH seen in 25% of cases versus 1.7% of controls (p < 0.001). Free T3 and free T4 levels were significantly lower in cases (fT3: 1.94 ± 0.58 vs. 2.81 ± 0.83 pg/mL, p < 0.001; fT4: 1.21 ± 0.39 vs. 1.35 ± 0.31 ng/dL, p = 0.032). Low fT3 and fT4 levels were more frequent among cases (21.7% and 20%, respectively) compared to controls (6.7% and 1.7%, p = 0.020 and p < 0.001). These findings indicate a higher prevalence of thyroid dysfunction, particularly hypothyroidism, among non-alcoholic liver disease patients compared to healthy individuals (Table 2 - see PDF). Liver function markers were significantly deranged in NAFLD patients. SGPT (52.51 ± 35.61 vs. 23.5 ± 9.61 U/L, p < 0.001), SGOT (45.3 ± 15.8 vs. 29.6 ± 9.7 U/L, p < 0.001), total bilirubin (6.47 ± 7.5 vs. 0.38 ± 0.22 mg/dL, p < 0.001), direct bilirubin (3.76 ± 4.33 vs. 0.19 ± 0.15 mg/dL, p < 0.001) and indirect bilirubin (2.71 ± 3.84 vs. 0.19 ± 0.15 mg/dL, p < 0.001) were all significantly elevated in cases. Lipid profile showed higher total cholesterol (202.5 ± 38.1 vs. 180.3 ± 32.4 mg/dL, p = 0.008), LDL-c (124.7 ± 30.5 vs. 106.8 ± 28.1 mg/dL, p = 0.012) and triglycerides (186.4 ± 55.2 vs. 132.7 ± 42.8 mg/dL, p < 0.001), with lower HDL-c in cases (38.5 ± 8.7 vs. 47.2 ± 9.1 mg/dL, p < 0.001). Fasting glucose (126.8 ± 28.5 vs. 98.4 ± 14.7 mg/dL, p < 0.001) and HbA1c (6.8 ± 1.5% vs. 5.4 ± 0.6%, p < 0.001) were also significantly higher in cases. Hemoglobin was lower in cases (9.26 ± 2.86 vs. 11.67 ± 2.25 g/dL, p < 0.001), indicating more frequent anemia. MCV and MCH showed no significant differences between groups. These findings suggest that non-alcoholic liver disease is associated with higher prevalence of metabolic risk factors, including central obesity, dyslipidaemia, hyperglycaemia and liver enzyme elevation, compared to controls (Table 3 - see PDF). The mean age was significantly lower in Grade 1 fatty liver (34.3 ± 8.94 years) compared to Grades 2 (48.1 ± 14.57 years) and 3 (42.17 ± 10.72 years) (p < 0.001). Male predominance was consistent across all grades (p = 0.72). BMI and waist circumference increased with disease severity (BMI: 25.6 ± 3.9, 28.2 ± 4.2, 30.3 ± 4.7 kg/^2^; waist: 91.5 ± 8.4, 98.2 ± 9.7, 103.4 ± 10.2 cm; p < 0.001). Obesity was most frequent in Grade 3 (75%). Diabetes (22.2%, 42.9%, 58.3%; p = 0.027) and hypertension (29.6%, 47.6%, 66.7%; p = 0.016) also increased with fatty liver grade. Smoking was more common in higher grades but not statistically significant (p = 0.154) (Table 4 - see PDF). Thyroid dysfunction increased significantly with fatty liver severity (p < 0.001). Normal thyroid function decreased from 96.3% in Grade 1 to 76.2% in Grade 2 and 25% in Grade 3. Overt hypothyroidism increased from 3.7% (Grade 1) to 14.3% (Grade 2) and 66.7% (Grade 3), while subclinical hypothyroidism appeared in 9.5% (Grade 2) and 8.3% (Grade 3). Mean TSH levels progressively increased (2.83 ± 1.35, 4.27 ± 2.12 and 13.08 ± 8.91 µIU/mL; p < 0.001), with high TSH (> 5.1 µIU/mL) in 3.7%, 23.8% and 75%, respectively. fT3 and fT4 levels declined with severity (fT3: 2.18 ± 0.4, 1.99 ± 0.48, 1.3 ± 0.63 pg/mL, p < 0.001; fT4: 1.33 ± 0.3, 1.2 ± 0.36, 0.98 ± 0.52 ng/dL, p = 0.030), with low levels more frequent in higher grades (Table 5 - see PDF). Liver function tests worsened with fatty liver severity. SGPT (24.35 ± 10.79, 59.63 ± 8.61, 103.41 ± 39.04 U/L; p < 0.001), SGOT (38.2 ± 12.5, 46.8 ± 14.3, 54.2 ± 17.1 U/L; p = 0.002) and total bilirubin (0.7 ± 0.43, 7.4 ± 4.49, 17.82 ± 6.41 mg/dL; p < 0.001) all increased progressively from Grades 1 to 3, with similar trends in direct and indirect bilirubin (p < 0.001). Lipid profile showed increasing total cholesterol (p = 0.012), LDL-c (p = 0.018), triglycerides (p = 0.005) and declining HDL-c (p < 0.001) with higher grades. Fasting glucose (p = 0.006) and HbA1c (p = 0.008) increased significantly with severity. Hemoglobin levels decreased with fatty liver grade (p = 0.011), while MCV and MCH showed no significant differences (Table 6 - see PDF). Overall, the findings indicate a significant association between increasing fatty liver severity and metabolic derangements, including worsening glycaemic control, dyslipidaemia and central obesity and elevated liver enzymes, suggesting a progressive metabolic burden with advancing fatty liver disease.

## Discussion:

In the present study, the mean age of NAFLD patients (40.7 ± 12.93 years) was comparable to controls (p = 0.217), similar to Parikh *et al.* [10] (44.3 ± 3.2 years vs. 41.6 ± 3.89 years, p > 0.05). However, Dhaliwal *et al.* [11] (54.81 ± 17.29 years) reported older populations, suggesting age-related progression of NAFLD. A male predominance (76.7%) was noted in our study, aligning with Mohanty *et al.* [12] (63.3%) In the present study, BMI was significantly higher in NAFLD cases (27.8 ± 4.6 kg/^2^) than in controls (24.5 ± 3.8 kg/^2^, p = 0.002), with obesity (BMI ≥ 25 kg/^2^) more prevalent in cases (50% vs. 33.3%). Waist circumference was also significantly higher (96.8 ± 10.5 cm vs. 85.4 ± 9.2 cm, p < 0.001). Mahashabde *et al.* [13] observed higher BMI in NAFLD cases (25.82 ± 4.79) compared to non-NAFLD (24.97 ± 4.34), with the highest prevalence in the 25-29.9 kg/^2^ range (37.2%). These findings reinforce the role of obesity and central adiposity in NAFLD pathogenesis, primarily through insulin resistance and hepatic fat accumulation. In the present study, diabetes (36.7% vs. 13.3%, p = 0.005) and hypertension (43.3% vs. 20%, p = 0.012) were significantly more prevalent in NAFLD cases compared to controls. Eshraghian *et al.* [14] identified metabolic syndrome, obesity, hypertension, diabetes and elevated liver enzymes as significant risk factors for NAFLD. In the present study, among NAFLD patients, 45% had Grade 1, 35% had Grade 2 and 20% had Grade 3 fatty livers. These findings suggest most patients presenting with early-stage NAFLD.

The present study found a significantly higher prevalence of thyroid dysfunction in NAFLD patients compared to controls (p < 0.001), with overt and subclinical hypothyroidism affecting 20% and 5% of cases, respectively. TSH levels were significantly elevated in NAFLD patients (5.38 ± 5.71 µIU/mL vs. 2.73 ± 1.47 µIU/mL, p < 0.001), while fT3 and fT4 levels were lower, suggesting an association between hypothyroidism and NAFLD. These findings align with systematic evidence of Eshraghian *et al.* [6] of 11 studies (n=12,924), which reported hypothyroidism prevalence ranging from 15.2% to 36.3% in NAFLD/NASH patients. Our observed TSH elevation (5.38 ± 5.71 µIU/mL vs. 2.73 ± 1.47 µIU/mL, p < 0.001) and reduced fT3 and fT4 levels support recent meta-analytic evidence. A comprehensive meta-analysis by Li *et al.* [8] provided dose-response evidence showing elevated free T3 significantly increases NAFLD risk (OR=1.580, 95% CI 1.370-1.830, p<0.001), while higher free T4 demonstrates a protective effect, with each 1 ng/dL increase above 1.019 ng/dL reducing NAFLD risk by 10.56%. Pagadala *et al.* [15] found a higher hypothyroidism prevalence in NAFLD (21% vs. 9.5%, p = 0.01), with hypothyroidism increasing NAFLD risk (OR = 2.1, 95% CI 1.1-3.9, p = 0.02). He *et al.* [16] conducted a meta-analysis of 13 studies, confirming a significant association between NAFLD and hypothyroidism (OR = 1.52, 95% CI 1.24-1.87, p < 0.001). Adjusted ORs remained significant for overt (1.81, 95% CI 1.30-2.52, p < 0.001) and subclinical hypothyroidism (1.63, 95% CI 1.19-2.24, p = 0.002). Mantovani *et al.* [17] confirmed primary hypothyroidism as an independent NAFLD risk factor (OR = 1.42, 95% CI 1.15-1.77). The present study found a significant association between thyroid dysfunction and fatty liver severity. Normal thyroid function declined with increasing fatty liver grades, while overt and subclinical hypothyroidism progressively increased. Overt hypothyroidism was seen in 3.7% of Grade 1, 14.3% of Grade 2 and 66.7% of Grade 3 cases, while subclinical hypothyroidism was noted in 9.5% of Grade 2 and 8.3% of Grade 3 cases. Serum TSH levels were significantly higher, whereas fT3 and fT4 levels were markedly lower in patients with more severe fatty liver. These findings align with previous studies that have examined the relationship between thyroid function and NAFLD. The inverse association between free T4 levels and fatty liver severity was evident, with 9%, 21% and 64% of patients in Grades 1, 2 and 3, respectively, exhibiting low fT4 (p = 0.000), consistent with findings from Ittermann *et al.* [18], Xu *et al.* [19] and Chung *et al.* [20]. Bano *et al.* [21] confirmed that higher free T4 levels reduce NAFLD risk, whereas elevated TSH levels increase the risk of significant fibrosis. These findings underscore the strong association between thyroid dysfunction and NAFLD progression, highlighting the need for routine thyroid function assessments in fatty liver patients. Further research into thyroid hormones' role in hepatic metabolism could enhance our understanding of NAFLD pathophysiology.

The pathophysiological basis for the thyroid-NAFLD association involves multiple interconnected mechanisms. Efstathiadou *et al.* [22] emphasized that NAFLD shares common clinical features with hypothyroidism, including obesity, insulin resistance, and dyslipidaemia all significantly more prevalent in our NAFLD cases. Gariani *et al.* [7] described how elevated TSH directly acts on hepatocyte TSH receptors to upregulate sterol regulatory element-binding transcription factor 1 (SREBP-1c), increasing triglyceride synthesis and hepatic accumulation. Low thyroid hormone levels impair lipid metabolism through reduced lipolysis and fatty acid oxidation, leading to hepatic fat accumulation. Up to 90% of hypothyroid patients exhibit dyslipidemia characterized by elevated triglycerides due to increased hepatic fatty acid esterification and reduced lipoprotein lipase activity. Additionally, hypothyroidism significantly impacts glucose metabolism by reducing insulin sensitivity, impairing hepatic glucose production and peripheral glucose uptake, ultimately contributing to insulin resistance a key driver of NAFLD pathogenesis. Recent mechanistic insights include adipocytokine dysregulation, particularly altered leptin levels that can promote hepatic collagen production and exacerbate insulin resistance. Oxidative stress represents another pathway, with hypothyroidism-associated mitochondrial dysfunction leading to increased reactive oxygen species production, lipid peroxidation, and hepatocyte injury. In the present study, liver function markers were significantly altered in NAFLD patients, with markedly elevated SGPT, SGOT and bilirubin levels compared to controls. These findings align with the established association between NAFLD and dyslipidemia, which plays a crucial role in hepatic fat accumulation and disease progression. The altered lipid profile in NAFLD is primarily driven by insulin resistance, which promotes hepatic lipid synthesis and impairs lipid clearance, further exacerbating hepatic steatosis. In the present study, haemoglobin levels were significantly lower in NAFLD patients (9.26 ± 2.86 g/dL) compared to controls (11.67 ± 2.25 g/dL, p < 0.001). The high prevalence of anaemia in NAFLD may be attributed to chronic inflammation, which disrupts erythropoiesis, as well as potential deficiencies in iron metabolism associated with liver dysfunction. Hepatic inflammation and altered cytokine profiles, particularly elevated interleukin-6 (IL-6), may contribute to anaemia by inducing hepcidin production, leading to impaired iron availability and utilization [23].

## Conclusion:

Thyroid dysfunction, particularly elevated TSH and reduced fT3/fT4 levels, was significantly associated with increasing severity of NAFLD. The correlation between thyroid abnormalities and worsening liver function suggests a contributory role of thyroid status in disease progression. These findings underscore the potential role of thyroid evaluation in the clinical assessment and management of NAFLD.

## Source of support:

No
